# Efficacy, safety, and PD-L1–defined outcomes of neoadjuvant immune checkpoint inhibitors in early-stage triple-negative breast cancer: a systematic review and meta-analysis

**DOI:** 10.3389/fimmu.2026.1933897

**Published:** 2026-08-12

**Authors:** Chang Ma, Hoon Koon Teoh, Delai Qiu, Yingnan Zhang, Shubao Yang, Yang Zhao, Chen Zhao, Hooi Tin Ong

**Affiliations:** 1 School of Public Health, Jilin Medical University, Jilin City, Jilin, China; 2 Department of Preclinical Sciences, M. Kandiah Faculty of Medicine and Health Sciences, Universiti Tunku Abdul Rahman, Sg Long, Kajang, Selangor, Malaysia; 3 School of Basic Medical, Jilin Medical University, Jilin, China; 4 Department of Emergency and Intensive Medicine, No. 965 Hospital of PLA Joint Logistic Support Force, Jilin City, Jilin, China

**Keywords:** immune checkpoint inhibitors, meta-analysis, neoadjuvant therapy, pathological complete response, PD-L1, triple-negative breast cancer

## Abstract

**Background:**

Neoadjuvant immune checkpoint inhibitors combined with chemotherapy improve pathological complete response in early-stage triple-negative breast cancer, but the consistency of benefit, role of PD-L1 expression, and immune-related toxicity remain clinically relevant.

**Methods:**

PubMed, Embase, and the Cochrane Central Register of Controlled Trials were searched from inception to June 30, 2026, for randomized trials comparing neoadjuvant immune checkpoint inhibitors plus chemotherapy with chemotherapy alone or placebo plus chemotherapy in early-stage triple-negative breast cancer. The primary efficacy analysis was restricted to phase III trials. Risk ratios and 95% confidence intervals were pooled using a random-effects model. Risk of bias and certainty of evidence were assessed using RoB 2 and GRADE.

**Results:**

Four phase III trials including 3,377 patients were included in the primary efficacy synthesis. Neoadjuvant immune checkpoint inhibitors plus chemotherapy significantly increased pathological complete response compared with control treatment (risk ratio, 1.27; 95% confidence interval, 1.19–1.36; P < 0.001), with no statistical heterogeneity. Benefit was observed in both PD-L1-positive tumors (risk ratio, 1.32; 95% confidence interval, 1.18–1.48) and PD-L1-negative tumors (risk ratio, 1.45; 95% confidence interval, 1.22–1.72). Grade ≥ 3 treatment-related adverse events were not significantly increased (risk ratio, 1.06; 95% confidence interval, 0.98–1.13), whereas immune-related adverse events were more frequent with immune checkpoint inhibitors (risk ratio, 3.52; 95% confidence interval, 1.88–6.57).

**Conclusions:**

Neoadjuvant immune checkpoint inhibitors plus chemotherapy improve pathological complete response in early-stage triple-negative breast cancer, including across PD-L1-defined subgroups, but increase immune-related toxicity. PD-L1 expression alone appears insufficient for treatment selection.

**Systematic review registration:**

https://www.crd.york.ac.uk/PROSPERO/recorddashboard#, identifier CRD420261412366.

## 1 Introduction

Triple-negative breast cancer (TNBC) is an aggressive breast cancer subtype, characterized by a lack of estrogen receptor, progesterone receptor, and human epidermal growth factor receptor 2 (HER2) expression (1–3). Historically, neoadjuvant chemotherapy (NACT) has been the cornerstone of treatment for early-stage, high-risk TNBC. Achieving a pathological complete response (pCR) following NACT is a well-established surrogate marker for improved long-term outcomes, including event-free survival (EFS) and overall survival (OS) (4–6). However, despite the optimization of anthracycline- and taxane-based regimens, a substantial proportion of patients fail to achieve pCR and subsequently face a high risk of early distant recurrence and mortality (7–9).

The treatment landscape for early-stage TNBC has changed substantially by the integration of immune checkpoint inhibitor (ICIs) (10). The rationale for this approach stems from the inherently higher immunogenicity of TNBC, characterized by elevated tumor mutational burden and abundant tumor-infiltrating lymphocytes (TILs) compared to other breast cancer subtypes (11, 12). Landmark trials, most notably KEYNOTE-522, have demonstrated that the addition of pembrolizumab to NACT significantly improves both pCR rates and long-term EFS, establishing chemo-immunotherapy as the new standard of care (13).

Despite these clinical benefits, the broad application of ICIs in early-stage TNBC presents a clinical challenge (14, 15). Immune-related adverse events (irAEs), such as endocrinopathies (e.g., thyroid dysfunction, adrenal insufficiency), are often irreversible and carry lifelong implications for patients with potentially curable early-stage disease (16). Consequently, there is a clinical need to identify reliable biomarkers that can accurately select patients who truly require ICIs, thereby sparing others from unnecessary toxicity and financial burden (17). While programmed death-ligand 1 (PD-L1) expression is a validated predictive biomarker in the metastatic setting, its utility in early-stage TNBC remains highly controversial (18, 19). Preliminary data suggest that the neoadjuvant benefit of ICIs might extend to PD-L1-negative populations, indicating that a single biomarker is insufficient to capture the complex interplay between the tumor and the host immune system (20). Instead, a broader understanding of the tumor immune microenvironment (TIME) is required to guide treatment tailoring (21).

We therefore conducted a systematic review and meta-analysis of randomized trials to evaluate the efficacy and safety of neoadjuvant ICIs plus chemotherapy in early-stage TNBC, with particular emphasis on PD-L1-defined treatment effects, immune-related toxicity, and implications for integrated biomarker strategies.

## 2 Methods

### 2.1 Search strategy and selection criteria

This systematic review and meta-analysis was conducted in accordance with the PRISMA 2020 statement. The protocol was registered in PROSPERO under the registration number CRD420261412366. A comprehensive literature search was performed across PubMed, Embase, and the Cochrane Central Register of Controlled Trials from database inception to June 2026. Search terms included combinations of “triple-negative breast cancer”, “immune checkpoint inhibitors”, “neoadjuvant therapy”, and specific drug names (e.g., pembrolizumab, atezolizumab). We also manually screened abstracts from major oncology conferences, including the American Society of Clinical Oncology (ASCO) and the European Society for Medical Oncology (ESMO), to identify the most up-to-date clinical trial results.

Studies were considered eligible based on the following PICOS criteria:

1. Patients: Individuals with early-stage, non-metastatic TNBC.
2. Intervention: Neoadjuvant ICIs combined with standard chemotherapy.
3. Comparison: Neoadjuvant chemotherapy plus placebo or chemotherapy alone.
4. Outcomes: Pathological complete response (pCR, defined as ypT0/Tis ypN0) reported as a primary or co-primary endpoint.
5. Study design: Phase II or III randomized controlled trials (RCTs).

To ensure clinical homogeneity in the primary efficacy synthesis, trials incorporating a window-of-opportunity design or monotherapy run-in phase before concurrent chemo-immunotherapy were excluded from the primary quantitative efficacy analysis. Studies lacking sufficient raw data for event-rate extraction were excluded from the corresponding quantitative analyses. For the primary efficacy synthesis, analyses were restricted to phase III RCTs. Eligible phase II randomized trials were considered for selected subgroup or safety analyses when endpoint-specific extractable data were available.

### 2.2 Data extraction and quality assessment

Two reviewers independently screened records, assessed full-text eligibility, and extracted data using a prespecified form. Extracted information included trial name, phase, publication year, sample size, disease stage, immune checkpoint inhibitor agent, chemotherapy backbone, treatment sequence, PD-L1 assay and cutoff, pCR definition, number of pCR events and randomized patients in each arm, and available safety outcomes. When multiple reports were available for the same trial, the most complete and updated dataset was used.

PD-L1 subgroup data were extracted as reported by each trial. Because PD-L1 positivity was defined using different assays and scoring algorithms, including 22C3 combined positive score and SP142 immune-cell scoring, subgroup findings were considered exploratory and were not used to infer comparative predictive performance between assays.

Risk of bias was assessed independently by two reviewers using the Cochrane Risk of Bias 2 tool. The domains assessed were bias arising from the randomization process, deviations from intended interventions, missing outcome data, outcome measurement, and selection of the reported result. Disagreements were resolved by discussion or by consultation with a third reviewer.

### 2.3 Statistical analysis

The principal summary measure was the risk ratio (RR) with its corresponding 95% confidence interval (CI) for pCR and adverse events. Given the anticipated clinical and methodological variations among the included trials, such as differences in chemotherapy backbones and specific ICI agents, a random-effects model (DerSimonian and Laird method) was *a priori* selected to pool the effect sizes. Statistical heterogeneity across studies was quantified using the Cochran’s Q test (with P < 0.10 considered statistically significant) and the I^2^ statistic (where I^2^ > 50% indicated substantial heterogeneity).

Subgroup analyses were pre-specified based on baseline PD-L1 expression status (positive vs. negative) and clinical parameters (e.g., nodal status). Safety outcomes were evaluated by pooling the incidence of Grade ≥ 3 treatment-related adverse events (TRAEs) and immune-related adverse events (irAEs). Sensitivity analyses were conducted using the leave-one-out method to assess the stability of the pooled results and the influence of individual studies. All statistical analyses were performed using R software (version 4.5.0, R Foundation for Statistical Computing). The certainty of evidence for key outcomes was assessed using the Grading of Recommendations Assessment, Development and Evaluation (GRADE) framework. Outcomes evaluated included overall pCR, pCR according to PD-L1 status, Grade ≥ 3 treatment-related adverse events, and immune-related adverse events. Evidence certainty was rated as high, moderate, low, or very low based on risk of bias, inconsistency, indirectness, imprecision, and potential publication bias. Publication bias was not formally assessed because fewer than ten studies were included in the primary efficacy synthesis.

## 3 Results

### 3.1 Study selection and patient characteristics

**Figure 1:**
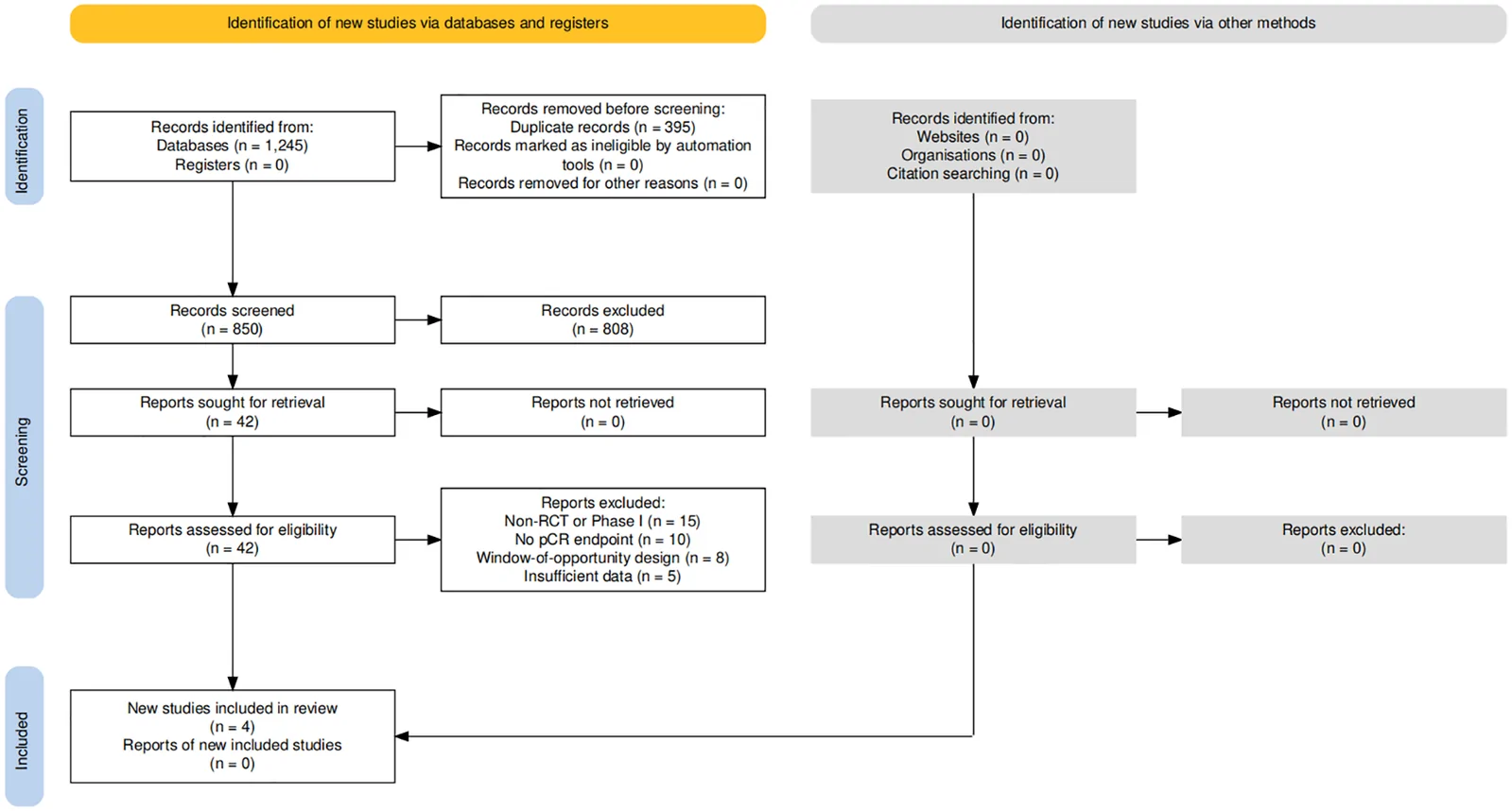
PRISMA flowchart. PRISMA flow diagram of study selection. The flow diagram summarizes the identification, screening, eligibility assessment, and inclusion of studies in this systematic review and meta-analysis. Four phase III randomized controlled trials (KEYNOTE-522, IMpassion031, NeoTRIP, and GeparDouze) were included in the primary efficacy synthesis. One additional phase II randomized trial (NCI-10013) was included in selected subgroup and safety analyses when endpoint-specific extractable data were available. PRISMA, Preferred Reporting Items for Systematic Reviews and Meta-Analyses; RCT, randomized controlled trial.

The initial literature search yielded 1,245 potentially relevant records. After removal of duplicates and screening of titles and abstracts, 42 full-text articles were assessed for eligibility. Four pivotal phase III RCTs-KEYNOTE-522, IMpassion031, NeoTRIP, and GeparDouze-were included in the primary efficacy synthesis. One additional phase II randomized trial, NCI-10013, was incorporated into selected subgroup and safety analyses when endpoint-specific extractable data were available (**Figure 1**).

The primary efficacy synthesis comprised 3,377 patients with early-stage TNBC, including 1,880 assigned to neoadjuvant ICIs plus chemotherapy and 1,497 assigned to chemotherapy with or without placebo. Baseline characteristics of the included randomized trials are summarized in **Table 1**, with NCI-10013 prespecified as contributing only to selected subgroup and safety analyses. Chemotherapy backbones varied across studies. Most phase III trials used sequential anthracycline- and taxane-based regimens, whereas NeoTRIP used an anthracycline-free carboplatin/nab-paclitaxel backbone. NCI-10013 also evaluated an anthracycline-free strategy but was considered separately from the primary phase III efficacy synthesis.

**Table 1:** Baseline characteristics of included randomized trials.

Study	Phase	ICI agent	Chemotherapy backbone	N, ICI/control	Median age, years	PD-L1 assay	PD-L1 positive %	Primary endpoint
KEYNOTE-522	III	Pembrolizumab	Carboplatin/paclitaxel → AC/EC	784/390	49	22C3 CPS ≥ 1	83%	pCR and EFS
IMpassion031	III	Atezolizumab	Nab-paclitaxel → AC	165/168	51	SP142 IC ≥ 1%	46%	pCR
NeoTRIP	III	Atezolizumab	Carboplatin/nab-paclitaxel	138/142	50	SP142 IC ≥ 1%	56%	EFS and pCR
GeparDouze	III	Atezolizumab	Carboplatin/paclitaxel → EC	793/797	50	SP142 IC ≥ 1%	58%	pCR and EFS
NCI-10013	II	Pembrolizumab	Anthracycline-free backbone	45/16	NR	NR	NR	pCR

NCI-10013 was not included in the primary phase III efficacy synthesis but contributed to selected subgroup and safety analyses when endpoint-specific extractable data were available. AC, doxorubicin plus cyclophosphamide; CPS, combined positive score; EC, epirubicin plus cyclophosphamide; EFS, event-free survival; ICI, immune checkpoint inhibitor; IC, immune cells; NR, not reported; pCR, pathological complete response.

Risk-of-bias assessment indicated that most included randomized trials had low risk of bias across key domains. Some concerns were noted for selected studies, mainly because of differences in trial design, endpoint hierarchy, maturity of selected outcomes, and incomplete reporting of some subgroup-specific data. No study was judged to be at high overall risk of bias (**Supplementary Table 1**).

### 3.2 Efficacy: overall and PD-L1 stratified pCR

**Figure 2:**
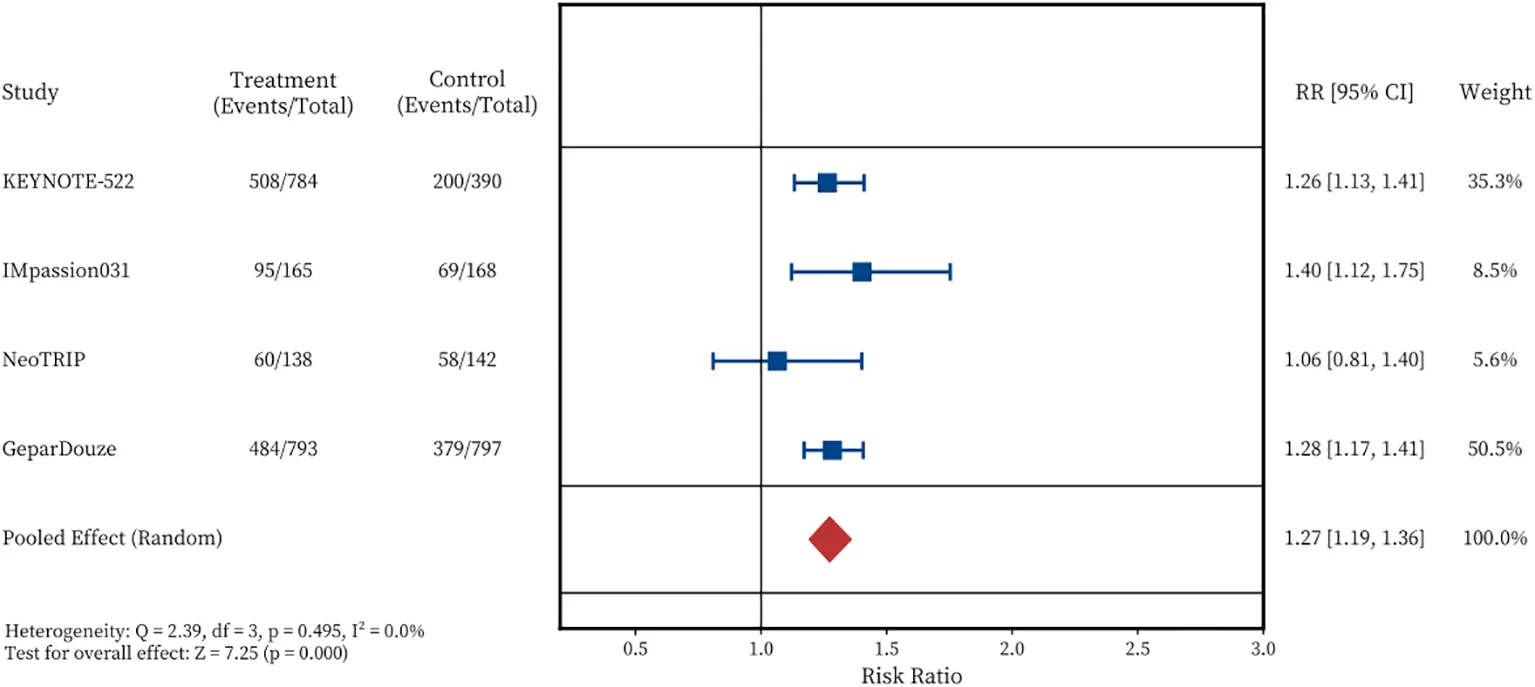
Overall efficacy (pCR) forest plot. Overall effect of neoadjuvant immune checkpoint inhibitors plus chemotherapy on pathological complete response. Forest plot showing the risk ratio for pathological complete response in patients with early-stage triple-negative breast cancer treated with neoadjuvant immune checkpoint inhibitors plus chemotherapy compared with chemotherapy alone. The primary efficacy synthesis included four phase III randomized controlled trials. Study-specific risk ratios are shown with 95% confidence intervals, and the pooled estimate was calculated using a random-effects model. Squares represent individual study estimates, with size reflecting study weight; horizontal lines indicate 95% confidence intervals; and the diamond represents the pooled estimate. CI, confidence interval; ICI, immune checkpoint inhibitor; pCR, pathological complete response; RR, risk ratio.

The addition of ICIs to neoadjuvant chemotherapy significantly improved the overall pCR rate compared with chemotherapy alone. Across the primary phase III trials, the pooled RR for pCR was 1.27 (95% CI, 1.19–1.36; P < 0.001), with no evidence of statistical heterogeneity (I^2^ = 0.0%, P = 0.495) (**Figure 2**).

**Figure 3:**
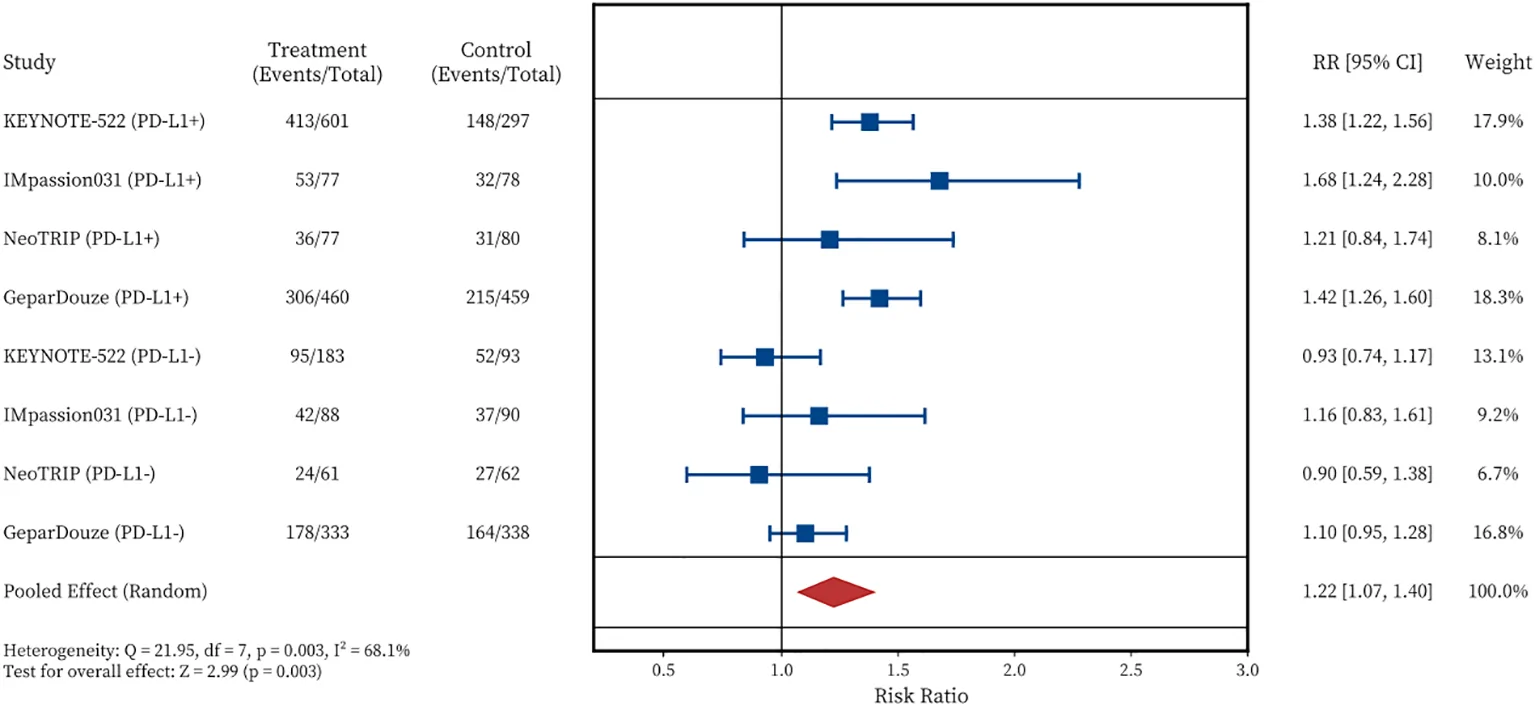
Stratified analysis by PD-L1 status. Pathological complete response according to PD-L1 expression status. Forest plot showing the association between neoadjuvant immune checkpoint inhibitors plus chemotherapy and pathological complete response stratified by baseline PD-L1 status. PD-L1-positive and PD-L1-negative subgroups were defined according to the criteria reported in each trial, including 22C3 combined positive score and SP142 immune-cell scoring where applicable. Study-specific risk ratios are shown with 95% confidence intervals, and pooled estimates were calculated using a random-effects model. CI, confidence interval; CPS, combined positive score; IC, immune cells; ICI, immune checkpoint inhibitor; pCR, pathological complete response; PD-L1, programmed death-ligand 1; RR, risk ratio.

In the PD-L1-positive subgroup, ICIs plus chemotherapy were associated with a higher likelihood of pCR than chemotherapy alone (RR = 1.32; 95% CI, 1.18–1.48; P < 0.001). A significant pCR improvement was also observed in the PD-L1-negative subgroup (RR = 1.45; 95% CI, 1.22–1.72; P < 0.001) (**Figure 3**). Because PD-L1 assays and subgroup definitions differed across trials, these estimates should be interpreted as evidence of preserved benefit across PD-L1 strata rather than as a direct comparison of treatment effect magnitude between PD-L1-positive and PD-L1-negative disease.

**Figure 4:**
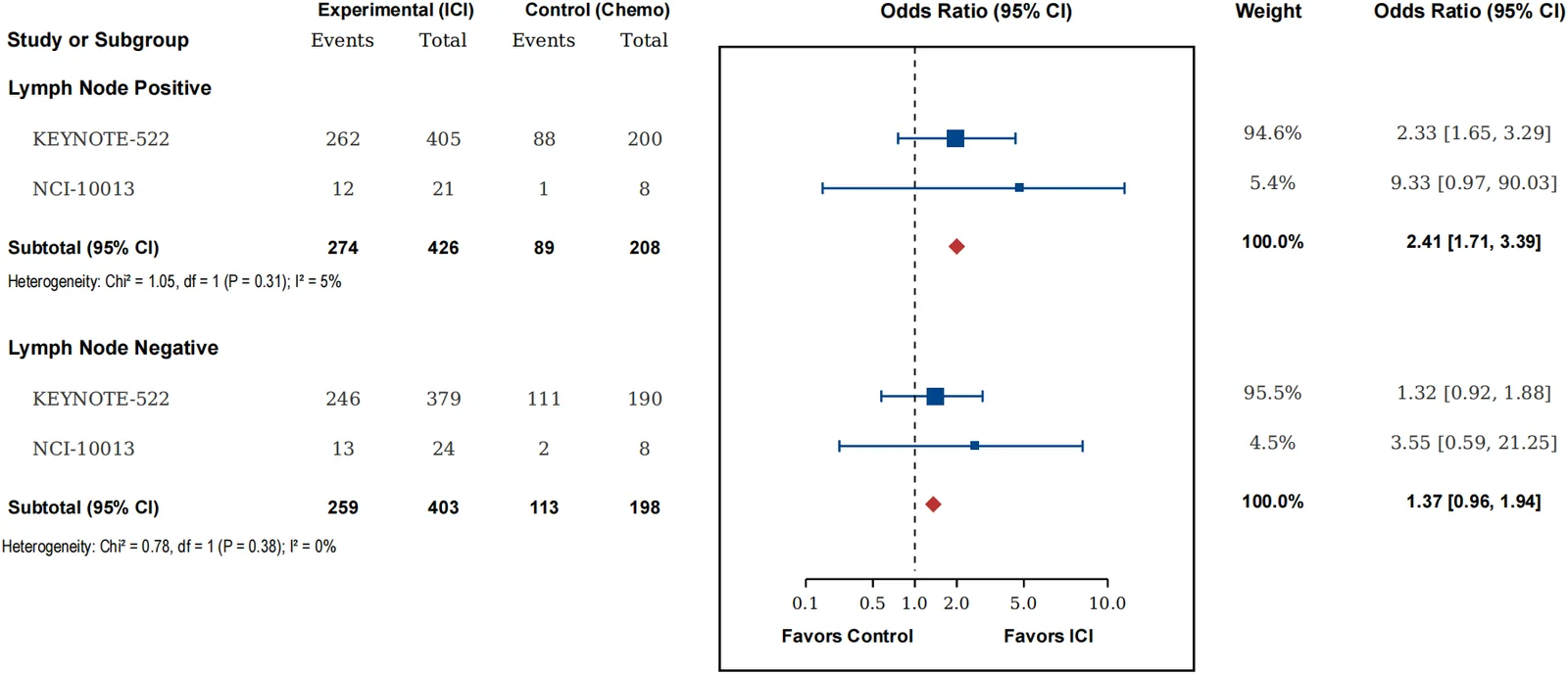
Clinical subgroups forest plot. Clinical subgroup analysis of pathological complete response according to nodal status. Forest plot showing the effect of neoadjuvant immune checkpoint inhibitors plus chemotherapy on pathological complete response according to baseline nodal status. Pooled risk ratios were estimated separately for patients with node-positive and node-negative disease using a random-effects model. Study-specific estimates are shown with 95% confidence intervals where available. Squares represent individual study estimates, horizontal lines indicate 95% confidence intervals, and diamonds represent pooled subgroup estimates. CI, confidence interval; ICI, immune checkpoint inhibitor; pCR, pathological complete response; RR, risk ratio.

Subgroup analyses based on clinical variables, including nodal status, consistently favored the chemo-immunotherapy arm. The magnitude of benefit appeared greater among patients with node-positive disease (RR = 1.45; 95% CI, 1.18–1.77) than among node-negative patients (RR = 1.13; 95% CI, 0.98–1.30) (**Figure 4**). Study-level pCR outcomes and corresponding risk ratios are summarized in **Table 2**.

**Table 2:** Summary of pCR outcomes across the primary phase III trials.

Study	ICI agent	Chemotherapy backbone	pCR, ICI arm	pCR, control arm	RR for pCR (95% CI)	PD-L1 assay
KEYNOTE-522	Pembrolizumab	Carboplatin/paclitaxel → AC/EC	508/784 (64.8%)	200/390 (51.2%)	1.26 (1.13–1.41)	22C3 CPS ≥ 1
IMpassion031	Atezolizumab	Nab-paclitaxel → AC	95/165 (57.6%)	69/168 (41.1%)	1.40 (1.11–1.77)	SP142 IC ≥ 1%
NeoTRIP	Atezolizumab	Carboplatin/nab-paclitaxel	60/138 (43.5%)	58/142 (40.8%)	1.06 (0.81–1.40)	SP142 IC ≥ 1%
GeparDouze	Atezolizumab	Carboplatin/paclitaxel → EC	484/793 (61.0%)	379/797 (47.5%)	1.28 (1.17–1.41)	SP142 IC ≥ 1%
Pooled estimate	–	–	–	–	1.27 (1.19–1.36)	–

AC, doxorubicin plus cyclophosphamide; CI, confidence interval; CPS, combined positive score; EC, epirubicin plus cyclophosphamide; ICI, immune checkpoint inhibitor; IC, immune cells; pCR, pathological complete response; RR, risk ratio.

pCR was defined as ypT0/Tis ypN0 whenever available. The pooled estimate was calculated using a random-effects model restricted to the four primary phase III randomized controlled trials.

### 3.3 Safety profile: severe and immune-related adverse events

**Figure 5:**
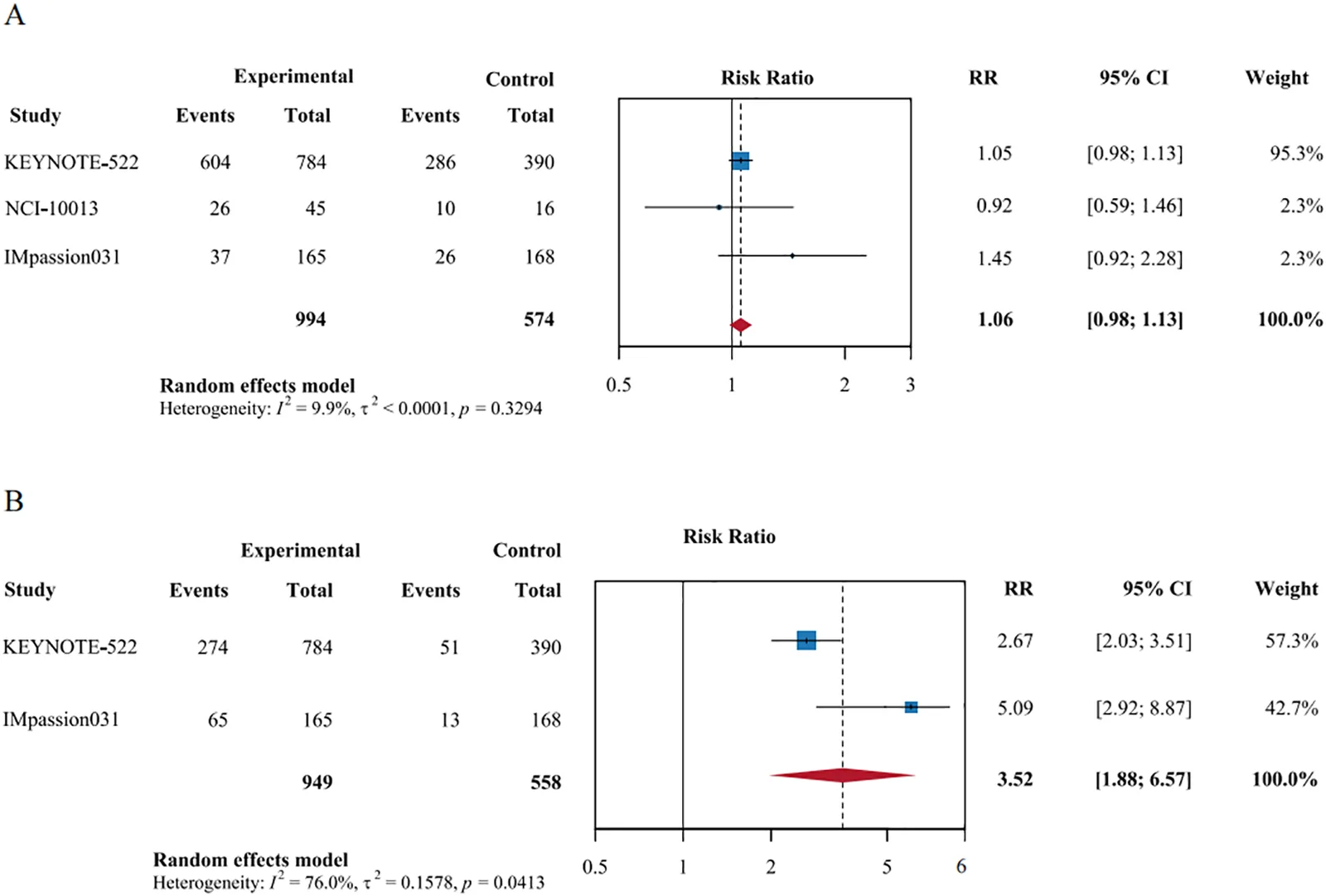
Safety profile forest plots. Safety profile of neoadjuvant immune checkpoint inhibitors plus chemotherapy. Forest plots showing the risk of adverse events associated with neoadjuvant immune checkpoint inhibitors plus chemotherapy compared with chemotherapy alone. **(A)** Risk ratio for Grade ≥ 3 treatment-related adverse events. **(B)** Risk ratio for immune-related adverse events of any grade. Safety analyses included trials with endpoint-specific extractable event-level data; therefore, the studies included for each safety endpoint may differ from those included in the primary efficacy synthesis. Study-specific risk ratios are shown with 95% confidence intervals, and pooled estimates were calculated using a random-effects model. Squares represent individual study estimates, horizontal lines indicate 95% confidence intervals, and diamonds represent pooled estimates. CI, confidence interval; ICI, immune checkpoint inhibitor; irAE, immune-related adverse event; RR, risk ratio; TRAE, treatment-related adverse event.

Safety analyses were conducted using trials with endpoint-specific extractable event data. The risk of Grade ≥ 3 treatment-related adverse events was slightly higher in the ICI arm than in the control arm, although the difference was not statistically significant (RR = 1.06; 95% CI, 0.98–1.13; P = 0.18) (**Figure 5A**).

In contrast, ICIs were associated with a marked increase in immune-related adverse events of any grade. The pooled RR for irAEs was 3.52 (95% CI, 1.88–6.57; P < 0.001), indicating a more than three-fold increase in risk compared with chemotherapy alone (**Figure 5B**). Leave-one-out sensitivity analyses showed that the pooled pCR estimate remained directionally consistent and statistically significant after sequential exclusion of individual studies (**Supplementary Figure 1**). Using the GRADE framework, the certainty of evidence was rated as high for the overall pCR benefit and moderate for PD-L1-defined subgroup outcomes and safety outcomes. Certainty was downgraded mainly because of differences in PD-L1 assay methodology, subgroup reporting, and variability in safety outcome reporting across trials (**Supplementary Table 2**).

## 4 Discussion

### 4.1 Summary of findings in the context of current practice

This meta-analysis summarizes contemporary randomized evidence on neoadjuvant ICIs in early-stage TNBC, with attention to PD-L1-defined subgroups and the tumor immune microenvironment. The primary efficacy synthesis showed that adding ICIs to NACT significantly improved pCR (RR = 1.27). This benefit was accompanied by increased immune-related toxicity, with a more than three-fold higher risk of irAEs.

The presence of pCR benefit in both PD-L1-positive and PD-L1-negative subgroups suggests that PD-L1 expression alone is unlikely to be sufficient for selecting patients for neoadjuvant immunotherapy in early-stage TNBC. These findings support broader biomarker strategies that incorporate immune contexture, tumor biology, and baseline clinical risk (22).

### 4.2 The paradox of PD-L1 and the imperative for TIME profiling

The finding that PD-L1-negative patients may still benefit from neoadjuvant ICIs raises important questions about the role of PD-L1 as a predictive biomarker in early-stage TNBC (23). In the metastatic setting, PD-L1 expression remains clinically relevant for selecting patients for pembrolizumab-based therapy. Its weaker discriminatory value in early-stage disease may reflect differences in tumor burden, antigen availability, host immune competence, and the integrity of tumor-draining lymph nodes (24). Several biological mechanisms may explain why patients with PD-L1-low or PD-L1-negative TNBC can still derive benefit from neoadjuvant ICIs (25). First, PD-L1 expression is spatially heterogeneous and dynamically regulated by inflammatory signals, particularly interferon-γ (26, 27); therefore, a single pretreatment biopsy may not fully capture PD-L1 status across the tumor or during treatment. Second, PD-L1 negativity does not necessarily indicate an immunologically cold tumor (28, 29). Some PD-L1-low tumors may still harbor abundant tumor-infiltrating lymphocytes (30), intact antigen-presentation machinery, and pre-existing T-cell-inflamed gene-expression programs, all of which may support sensitivity to immune checkpoint blockade (31, 32). Third, in the neoadjuvant setting, chemotherapy may augment antitumor immunity by inducing immunogenic cell death, increasing tumor-antigen release, promoting dendritic-cell priming, and enhancing cytotoxic T-cell infiltration (33, 34). These effects may convert a less inflamed tumor microenvironment into a more immune-responsive state, thereby reducing the dependence on baseline PD-L1 expression alone (35).

Accordingly, a single immunohistochemical marker is unlikely to capture the spatial and temporal complexity of the tumor immune microenvironment (36, 37). Early-stage TNBC is often characterized by higher immune infiltration and a less exhausted immune milieu than advanced disease (38–40). Exploratory analyses from adaptive neoadjuvant platforms, including I-SPY2, suggest that RNA-based immune signatures and broader measures of immune activation may better identify tumors that are sensitive to immune checkpoint blockade. These observations support a shift from single-marker selection toward integrated TIME profiling, incorporating PD-L1 status (41), stromal and intratumoral TILs, interferon-related signatures, antigen-presentation capacity, and other markers of adaptive immune activation.

### 4.3 The “*in situ* vaccine” hypothesis and the importance of timing

The contrast between the positive neoadjuvant trials and the negative adjuvant IMpassion030 study also highlights the potential importance of treatment timing. In the neoadjuvant setting, the primary tumor and draining lymph nodes remain *in situ*, providing a source of tumor antigens during chemotherapy-induced immunogenic cell death. Concurrent immune checkpoint blockade may enhance priming and expansion of tumor-specific T cells under these conditions (42).

By contrast, after surgical resection, the major antigen source has been removed, which may reduce the opportunity for effective immune priming. Although this hypothesis requires further validation, the available clinical evidence suggests that the neoadjuvant setting may be a particularly favorable therapeutic window for integrating ICIs in curable TNBC (43).

### 4.4 Optimizing the chemotherapy backbone: towards de-escalation

The optimal chemotherapy backbone for neoadjuvant chemo-immunotherapy remains unsettled. The KEYNOTE-522 regimen, which includes both platinum and anthracycline components, is now widely used, but its cumulative toxicity is substantial. De-escalation strategies are therefore clinically relevant, particularly for patients with lower-risk disease or favorable immune features (44, 45).

Although not part of the primary phase III efficacy synthesis, NCI-10013 provides hypothesis-generating evidence regarding anthracycline-free chemo-immunotherapy backbones (46). Avoiding anthracyclines could reduce long-term risks such as cardiotoxicity and therapy-related hematologic malignancies (47). Future trials should evaluate whether treatment intensity can be tailored according to baseline clinical risk, immune phenotype, and early response, rather than applying a uniform maximally intensive regimen to all patients.

### 4.5 Limitations

Several limitations should be acknowledged. First, the primary efficacy synthesis included only four phase III RCTs, although one additional phase II randomized trial contributed to selected subgroup and safety analyses (48). Second, this study was based on study-level rather than individual patient-level data, limiting the ability to adjust for patient-specific covariates such as tumor stage, nodal status, tumor-infiltrating lymphocyte density, germline BRCA status, and treatment completion (49). Third, clinical and methodological differences existed across trials, including the ICI agent used, chemotherapy backbone, treatment duration, and PD-L1 assay methodology (50). In particular, PD-L1 positivity was defined using different platforms and scoring systems, including 22C3 CPS and SP142 immune-cell scoring, which limits direct comparability between studies. Fourth, safety reporting was not fully standardized, and safety meta-analyses were restricted to trials with endpoint-specific extractable event data (51).

Publication bias could not be reliably assessed because of the small number of studies included in the primary efficacy synthesis (52). The GRADE assessment indicated moderate certainty for PD-L1-defined subgroup and safety outcomes, largely because of assay heterogeneity and non-uniform safety reporting (53). Finally, although pCR is an accepted surrogate endpoint in early-stage TNBC, mature EFS and OS data are not yet uniformly available across all included trials (54).

## 5 Conclusion

In this systematic review and meta-analysis of randomized trials, neoadjuvant ICIs plus chemotherapy significantly improved pCR in patients with early-stage TNBC, with benefit observed across available PD-L1-defined subgroups. However, this efficacy gain was accompanied by a substantial increase in irAEs, underscoring the need for careful toxicity monitoring in the curative-intent setting. PD-L1 expression alone appears insufficient for patient selection, and future studies should prioritize integrated biomarker models incorporating clinical risk, tumor immune contexture, and dynamic response markers to optimize benefit-risk balance and guide rational de-escalation.

## Data Availability

The original contributions presented in the study are included in the article/**Supplementary Material**. Further inquiries can be directed to the corresponding authors.

## Supplementary material

Supplementary Table 1 Risk-of-bias assessment using the Cochrane RoB 2 tool.

Supplementary Table 2 GRADE evidence profile for key outcomes.

Supplementary Figure 1 Sensitivity analysis. Leave-one-out sensitivity analysis for the primary efficacy outcome. Leave-one-out sensitivity analysis assessing the robustness of the pooled effect of neoadjuvant immune checkpoint inhibitors plus chemotherapy on pathological complete response. Each analysis was performed by sequentially excluding one study from the primary efficacy synthesis and recalculating the pooled risk ratio using a random-effects model. The pooled estimates remained directionally consistent and statistically significant across all iterations. CI, confidence interval; ICI, immune checkpoint inhibitor; pCR, pathological complete response; RR, risk ratio.
